# An audit of corporate decarbonisation ambition against low carbon futures

**DOI:** 10.1038/s41598-025-20203-6

**Published:** 2026-01-06

**Authors:** Iain Weaver, Jesse F. Abrams, Jack Oliver, Nikolaos Dimakis, Andrew Parry, Timothy M. Lenton

**Affiliations:** 1 Global Systems Institute, University of Exeter, Northcote House, Streatham Campus, Exeter, EX4 4QE UK; 2 J O Hambro Capital Management, Level 3, 1 St James’s Market, London, SW1Y 4AH UK

**Keywords:** Attribution, Climate-change mitigation, Projection and prediction

## Abstract

Atmospheric greenhouse gas concentration has been steadily increasing since the 19th century, causing global warming. Despite efforts to reduce emissions, current projections anticipate a significant increase in global surface temperature by the end of the century, which may push us beyond a safe and just operating space. We must develop emissions pathways that consider renewable technology innovation, climate policy development and consumer behaviour change. In this manuscript we assess corporate emissions reduction ambition in the context of reduction pathways, and in doing so show that company reporting has reached a scale and quality that it can be used to supplement global emissions forecasting. We find emissions disclosures of target-setting companies to account for roughly 20% of global $\hbox {CO}_{2}$ emissions and 60% of global market capitalisation. Of these, we find near-term targets to be consistent with the aggressive reduction requirements of the divergent net-zero scenario published by the Network for Greening the Financial System, but commitments to 2050 are lacking. There is a large disparity between the total market capitalisation of disclosing companies and the total emissions they cover, a disconnect which we emphasise must be addressed by future policy.

## Introduction

Since around 1850, human activity has increased atmospheric concentrations of carbon dioxide and other greenhouse gases (GHGs), driving a rise in global surface temperature and associated climate change^1^. As of 2022, atmospheric $\hbox {CO}_2$ levels were nearly 50% higher than pre-industrial levels^2^, and global GHG emissions total approximately 53.8 $\hbox {GtCO}_2$e per year (6.8 t per person). These emissions are broadly distributed across sectors: 39% from power generation and fuel use, 21% from industry, 16% from transport, 12% from agriculture and land use, 7% from buildings, and 5% from waste^3^.

Although low carbon technologies are rapidly approaching cost parity with fossil-based incumbents^4,5^, current climate policy, including planned mitigation efforts, still puts the world on track for $\sim 2.7^{\circ }$C of warming by 2100^6–9^. This level of warming is expected to intensify already-visible climate impacts, such as extreme heat and ecosystem degradation, and raises the risk of triggering irreversible tipping points in Earth systems^10–12^.

Recent updates to 2030 emission reduction pledges (Nationally Determined Contributions) modestly improve the outlook, potentially limiting warming to $2.5^{\circ }$C if fully implemented^6^. However, this still exceeds the $2^{\circ }$C threshold widely considered necessary to avoid severe disruption to human and natural systems^13^. Achieving this goal will require rapid, economy-wide transformations in energy, industry, and transport^14–16^, with stronger commitments especially from developed countries.

Scientifically grounded emissions pathways are critical to this effort. Historical national GHG inventories are compiled by sources such as the Global Carbon Project^17^, Climate Watch^17^, and the European Environment Agency^18^. They form a foundation for both projections and scenario-based modelling. These projections help estimate the effect of climate policies, energy demand, and technology innovation^19–22^. However, these approaches often lack granularity on how market trends and climate policies are manifesting at the level of individual companies.

In this manuscript, we explore this emerging dimension by examining GHG emissions data and reduction targets disclosed by large companies responsible for a significant share of global emissions. While there is no universal reporting standard or centralised repository, some firms report climate data alongside financials, and a small number of providers aggregate this into proprietary datasets. We assess the current state of this corporate-level data landscape and its potential to supplement national and sectoral GHG tracking.

The Carbon Disclosure Project^23^ provides an avenue for companies to individually measure and report their environmental impacts, such as carbon emissions, water security, deforestation, via very broad, annual surveys. CDP is a not-for-profit institution that has built a network and a process for companies to voluntarily disclose their GHG emissions across scope 1, 2 and 3. Using standardised reporting and allowing for companies to use third party verification, the disclosures are compliant to the GHG Protocol standards (Details on the GHG Standards can be found here: https://ghgprotocol.org/standards). Institutional Shareholder Services (ISS)^24^ supplements this dataset with corporate disclosures via sustainability documents. Estimates of scope 3 emissions, those generated from downstream operation of a company’s product, suffer disproportionately from a lack of a consistent (both year-on-year and between industries) reporting methodology^25–28^, and further leads the disclosees to avoid setting targets for this metric. For these reasons, this manuscript focuses on scope 1 and 2 reporting which are themselves not free of any barriers to analysis. While they are somewhat standardised by the ISS and CDP frameworks, there is little to no oversight or validation in the data themselves. We are not forced to have absolute faith in the robustness of individual company reports, but we must confront the potential for errors in individual measurements of companies in high-intensity industries to dramatically skew the aggregate data. This issue is worsened because individual companies can independently modify their own measurement and assessment criteria. While there is guidance available, including by CDP directly, this is not finely tailored to every individual sector or industry, and ultimately there is no requirement to adhere to it.

These GHG emissions sources are supplemented by company GHG reduction targets. These are provided via CDP and directly from the Science Based Targets Initiative (SBTi)^29^. SBTi provides methods, guidance and ultimately certification for corporate GHG reduction targets which are consistent the Paris Agreement, as quantified by the IPCC^30–32^ AR6 which reports that, starting from 2020, the remaining carbon budget for a 50% chance of limiting warming to $1.5^{\circ }$C is approximately 500 GtCO${}_2$e. Many targets provided by CDP are certified as such, but we do not include this as a requirement to be included in the study. At the intersection of these data sources, there is the potential for a very granular view of the level of global engagement with climate issues. Indeed there is a substantial body of research in this area; a study of the GHG reduction progress of 115 companies reveals that targets based on renewable energy certificates are unlikely to drive renewable energy growth^33^; emission reduction strategies are dominated by reliance on renewable energy certificates in a sample of 102 companies^34^; and correlations can be drawn between an investigation of organisational performance and GHG reductions for 62 UK companies^35^.

In addition to this, there have been several studies into the relationships between company initiatives and GHG emissions across a range of contexts, including European firms^36,37^, the agricultural industry in China^38^, the global steel industry^39^, and firms in countries with sustained emissions reductions^40,41^. The purpose of this manuscript is to provide an in-depth view of the current state of GHG emissions accounting and target-setting in the global context of overall warming scenarios. Principally, we show that company reporting has now reached a scale and quality that it can be used as a serious supplement to other global monitoring schemes, although there remain significant blind-spots.

We provide an overview of the emissions and reduction ambitions through key sources on the company level. We first explore the coverage of these sources by company market capitalisation then by share of global emissions estimates. Market capitalisation (hereafter referred to simply as market cap), is the total value in USD of a company’s outstanding shares of stock, commonly used as a simple metric to measure the overall value of a company. We go on to assess company reduction ambitions against published reduction pathways provided by the Network for Greening the Financial Sector (NGFS)^42^, taking steps to address the multitude of challenges inherent in the data sets along the way.

## Methods

**Fig. 1:**
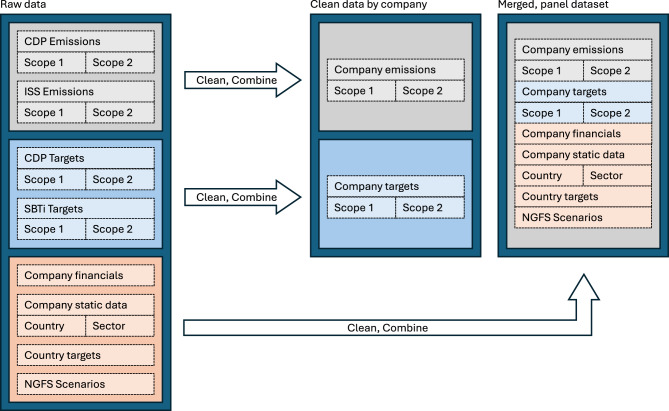
An overview of the data merging process. We combine emissions data from CDP, ISS, SBTi, Country-level targets, company static data such as country of registration and sector, and company financials. A significant amount of manual intervention is required to ensure companies with different identifiers are properly harmonised and not counted twice.

**Fig. 2:**
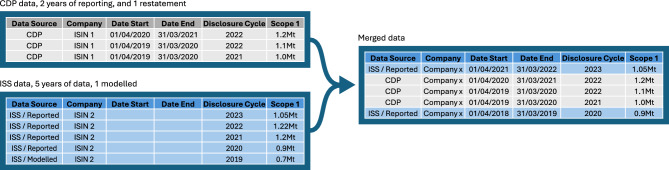
An illustration of the how the merging of emissions data from CDP and ISS takes place. From both CDP and ISS, we obtain data referring to a company identified by ISIN 1 for CDP, and ISIN 2 from ISS. From our internal reference data, we know that ISIN 1 and ISIN 2 refer to the same company, thus a merging of these datasets can take place. CDP data takes priority where available and modeled data provided by ISS is excluded from the dataset (Note that the resulting dataset does not include the data point for 2019, as this was an estimate from ISS). The date period of reference as inferred from the CDP disclosures.

This section outlines the primary data sources for disclosed company GHG emissions and reduction ambition, and details the approach used to filter inactive companies, map relevant financial data, and interpolate GHG reduction ambition. A high level overview of the interaction of data sources is provided by Figs. 1 and 2.

### Data

CDP and ISS are our sources for GHG emission data. Data provided by CDP in particular is enriched with a very large amount of contextual data; including emissions per business activity, facility, gas, company restatements and reasons for change. Notably, ISS do offer a category of ‘modelled emissions’ where financial data has been used to estimate emissions based on the behaviour of known peers. However we do not include these in any part of the analysis; their motivation is to capture as much of the public-traded world as possible, while ours is to assess the state of GHG reporting and target setting.

Emissions are typically partitioned into three non-intersecting scopes, the definition and boundary of which is defined in the GHG Protocol^43^, which provides a comprehensive framework of measuring and managing GHG emissions and emissions scope definitions. Further, ISO standards such as ISO140641^44^ and ISO140643^45^ shed light on how the emissions for each individual scope should be accounted for, and validated. This becomes an important factor to consider as with the growing interest in the carbon-footprint of companies, the data providers offering such data need not necessarily disclose what types of standards they are compliant with. The dataset which makes up the bulk of this manuscript, CDP, has defined that emissions data, and verification of those, should follow specific standards, making the information contained in their datasets significantly more reliable than others (accepted standards are enumerated on the CDP website under verification).

In spite of a push for robust standards in measuring GHG emissions such as the International Sustainability Standards Board, we should make clear that there is no uniform legislation for companies to disclose climate-related data across all jurisdictions. There are leaders in the space such as the EU with the Corporate Sustainability Reporting Directive (CSRD)^46^ where more detailed sustainability reporting requirements are introduced for EU companies, and non-EU companies with certain constraints on trading and turnover in the European market. The Taskforce on Climate-related Financial Disclosure (TCFD)^47^ framework is gradually being implemented in the UK across various organisational categories through an intricate network of legislation and regulations specific to different sectors. In the US, the SEC has proposed a new legislation that builds on the Taskforce on Climate-related Financial Disclosure recommendations^48^. Other major players are starting to introduce legislation addressing these issues, acknowledging their importance in the journey towards net-zero including China^49^, Japan^50^ and India^51^.

Scope 1 emissions are those from sources owned or controlled by a company such as from burning fuel in the companies fleet of vehicles. Scopes 2 and 3 are indirect emissions; those which are direct consequences of the activities of the company but occur from sources not owned or controlled by it. More specifically, scope 2 emissions cover the emissions from where the energy a company purchases and uses is produced, while scope 3 emissions encompass indirect emissions not directly generated by the company itself or its owned assets, but are instead a result of its activities throughout the entire value chain, both upstream and downstream.

Scope 2 emissions introduces a distinction between the ISS and CDP sources. Since 2015, CDP allows companies to distinguish between a *market-based* and *location-based* disclosures. Location-based emissions are the energy use of a company multiplied by the mean emissions of its location’s energy tariffs; that is countries with renewable sources as a large fraction of their grid diminish exposed companies location-based scope 2 emissions. Market-based emissions take into account distribution of energy tariffs a company uses. Where an energy provider gives a Renewable Energy Certificate, market-based emissions are based on this tariff which is typically lower than the corresponding location-based values (as previously noted, reliance on such a distinction forms a central component in some companies’ reduction ambition^34^). On the other hand, if such a certificate is not presented, tariffs are assumed to correspond to the uncertified subset of the domestic market, producing estimates greater than the location-based value. While it may be intuitive that this provides incentive for energy companies to shift their energy mix to fill this demand, there is mounting evidence that such an approach both underestimates the actual emissions produced under renewable certificates, and fails to drive increase in renewable energy supply^33,52–54^. Where available, we use location-based emissions values, otherwise relying on market-based estimates.

Scope 3 is an umbrella term for 15 categories of emissions, divided between upstream, such as waste generated in company activities, and downstream, including the use and disposal of sold products. These emissions cover all sources beyond the boundaries of scope 1 and scope 2 emissions. Accounting and disclosing emissions for scopes 1 and 2 are relatively straightforward, scope 3 requires a far more nuanced approach. For perspective, the summation of scope 3 disclosures for the 2021-2022 financial year alone accounts for approximately 211% of the 2022-2023 global GHG emissions, with the Electronic Technology sector accounting for 34% of this total. For this sector, scope 3 emissions are dominated by the use of its products. A first order approximation would be the mean energy consumption of the product, multiplied by the complement of renewable energy mix of the country it is operating in, *and multiplied by its expected lifespan*. The last factor here is critical to producing a meaningful estimate of scope 3 emissions, but it makes the result rather opaque to analysis; without granular information about the precise specification of products sold, and the number of units distributed over a time period of at least the lifespan of the product, we cannot make meaningful estimates of the annual contribution of these products, and concede that a thorough treatment of year-on-year scope 3 emissions is outside the remit of this work.

The number of companies with emissions disclosures captured by CDP and ISS are shown for scopes 1, 2 and 3 in Fig. 10c which shows almost all disclosing companies cover scopes 1 and 2, with 60% including scope 3. We should note here that CDP and ISS are not the only providers with emissions data although CDP disclosures typically form the bulk of the data redistributed by other providers, supplemented by their independent analytics and research. Such sources include MSCI, Sustainalytics (a part of Morningstar Inc.), Trucost (a part of S&P), however from our experience they fall short in terms of quality, depth and breadth of data as other studies have previously articulated^25–28^.

### Emission reduction targets

Target data is provided by CDP and supplemented by SBTi. There is a large intersection of these sources so care is taken to avoid duplication. As with emissions, CDP provide a broad context for individual targets and allows target setting outside the SBTi framework. Similarly, SBTi has a more frequent publishing schedule which can lead to new targets not yet incorporated by CDP. The number of companies contained in each dataset is shown in Fig. 10a

Corporate emission reduction targets are submitted alongside a *base year*, which is provided by the disclosing company as the year against which future emissions disclosures should be measured. Fig. 10b shows the distribution of target scopes. For the purposes of this study we reject scope 3 from stated targets, as we have noticed sparse and highly anomalous scope 3 reporting, making the implicit assumption that other scopes combined with scope 3 in a target will reduce proportionally. The majority of companies state targets combining scopes 1 and 2, although there is a significant subset which provide targets split on the individual scopes. In such cases, we estimate the aggregated reduction of these targets using the ratio of emissions at the base year.

The final component of a reduction target is the target dates, which predominantly fall into the corresponding company 2030, or 2050 disclosure dates, although recent events, including an address by the UN Secretary-General ahead of the latest synthesis report of the IPCC is likely to add many new targets for 2040^55^. We interpolate between a company’s base and target dates to produce an estimate of an intermediate target at 2030. For companies with a target before 2030, this will instead be a small extrapolation as we assume additional targets will emerge roughly consistent with previous company progress.

Many targets only address part of a company’s GHG emissions profile. Fig. 10b shows that 92% of companies which set targets address both scope 1 and 2 emissions which can be done either by submitting separate targets for scope 1 and 2, or combining them into a single target with an aggregated reduction. For targets on individual scopes, compute the influence of reductions on each scope to the company’s total emissions as a means to directly compare ambition against the joint targets. As well as limiting the scope of emissions covered by a corporate target, target statements coverage within scope can also be limited. Targets with a limited boundary can further muddy the water; a company can submit a comprehensive target while only covering a specific fraction of emissions in either scope. It is worth noting at this point that companies are motivated to submit more nuanced targets such as this in part because assessment bodies such as S&P Global Ratings ESG reward companies with comprehensive targets, that is targets covering some part of both scope 1 and 2.

Fortunately, this approach to target setting is not widespread. Of the 3,740 companies with at least one target, only 292 set only targets which do not fully cover the company’s scope 1 or 2 emissions. Of these, the majority (211) cover at least 50% of the emissions in the target scope. In all cases where the emissions covered are not 100%, we assume that firms abate only their stated boundary emissions, with no spill over into other parts of their operations. This makes our estimates conservative, since in practice abatement actions may reduce emissions beyond these boundaries the target reduction is adjusted to the fraction of all emissions in scope. Concretely, a 20% reduction on 50% of scope 2 emissions is read as a 10% target on all scope 2 emissions.

### Data curation

Data curation and preparation are a key stage of our process. Combining data sets from different data providers is always a challenging step, especially in cases where data differ. We begin by relating the CDP, SBTi and ISS identifiers with real companies, relying on company financial data via FactSet^56^, illustrated by Fig. 1. We curate and filter the data, taking care both to avoid double counting companies which exist in duplicate under different identifiers, and adding companies which did not exist between 2022-2023 to the audit. Within a dataset it is possible for a company to disclose emissions via multiple identifiers, either proprietary such as CDP’s internal identifier, or standardised such as ISIN used by ISS and SBTi. These identifiers are harmonised by linking to their financial data via Factset, providing a single identifier which can also be used to reject companies which no longer exist, defined here by those which have not reported financial information since 2020. However, within a single top-level identifier, it is possible for a company to have multiple identifiers especially in the case when company change legal structure; in such cases, we aim not to discard any data, but rather merge into a non-overlapping block of disclosures with priority given to data provided by CDP, illustrated in Fig. 2. Note that in this particular example, one of the entries in the disclosure cycle of 2022 was a restatement of its previous emissions. Further, ISS also provides emissions data since 2019, however note that the 2019 report is not based on the company’s disclosure but from ISS’s internal estimation model. In such a case, the CDP data is used over its entire domain 2021-2022, and ISS data fills in the missing data when possible. Fig. 10 shows the globally aggregated coverage of these datasets, along with the disclosed emissions scopes and targets.

### Reasons for change

Along with annual emissions, companies can also disclose the reasons for the differences since the previous disclosure via the CDP by selecting one of a small number of options. Incorporating these reasons into the analysis would introduce new challenges, which we do not directly address in this manuscript. The forms to convey reasons for change and the emissions disclosures themselves are independent, and as such, there is no cross-validation, nor any mechanism for companies to amend previous reasons for change when restating previous disclosure values. However, the stated reasons for the changes in the 2022-2023 interval are shown in Fig. 4, where totals are used aggregated only from companies with a difference less than 25% between the stated values in the disclosure and reasons for the change forms. This filtering removes a small number of extreme differences, likely introduced by typographical errors.

The dominant stated reason for emissions increase is changes in industry output, an intuitive result since year-on-year growth is the expectation for companies in any sector, and a corresponding increase in scope 1 and 2 emissions is expected. On the other hand, divestment and intentional emissions reductions are the dominant sources of emissions reductions. Divestment in this context is where a company sells its high GHG intensity assets, no longer accounting their emissions in subsequent emissions disclosures. We need not take a cynical perspective on this process to acknowledge that these large divestments will skew our view on the overall reduction ambition of companies as the target progress made by divesting in certain assets is limited and unlikely to be a part of a long-term strategy. This effect is managed along with unlabelled spurious changes in emissions in the following section.

### Restatements and anomalous changes

Companies are able to submit revised emissions numbers after the initial disclosure. There are numerous legitimate reasons to do so and CDP provides fields for a reporting company to provide context for the restatement. Parsing these fields in a nuanced way is tempting, however the remarks and comments are sparse, inconsistent, and often duplicated for all of a company’s disclosures rendering them not useful. The distribution of restatement factor is an interesting area of research. A central, long-tailed and roughly symmetrical distribution of restatement factor has a median change of 8% and a mean of 930% (owing to its characteristic fat-tails). The precise form of the distribution is compounded by the distinctive features of transcription errors, introducing many 10x and 1,000x errors, presumably due to errors transcribing single digits, and emissions units or thousands-separation characters. Restatements provide a framework for companies to correct such errors which we estimate to account for roughly 3% of all restatements, but the most recent disclosures (2023) have not had any opportunity to be addressed, and so we perform a cursory screening for such large factors. Where the most recent disclosure differs from the previous one by a factor of at least 100, we manually inspect all emissions for that company to establish cases where the difference is erroneous. While this method is likely to miss less egregious errors, we find this light touch to be sufficient to avoid any company skewing aggregated metrics.

Another key issue we address here arises where a company undergoes an apparently dramatic shift in their scope 1 emissions since the base year, sometimes by an order of magnitude or more. Such changes can be tied to world-events or even explained by the disclosing company themselves. As a recent example, the COVID-19 pandemic drove dramatic declines in emissions in multiple sectors. In the next years, global emissions rates returned to their baseline values somewhat faster than we might have hoped. Such shifts can also follow company restructuring, divesting in high-intensity assets or may appear for no notable reason at all as emissions measurements vary greatly due to inconsistent methodology. As a concrete example, a large electricity utility company based in Japan reduced its stated emissions in 2019 by transferring all thermal power production to a new company, a joint-venture with another electric utility. This resulted in a 90% reduction in the disclosed scope 1 emissions, a process known as ‘divestment’. While we do not question the veracity of such step-changes, we do question their overall contribution to the low carbon transition as they typically result from divestment or similar processes. In the case of CDP data, this is often reported explicitly. We handle this by identifying step changes between emissions measurements with a time series analysis; identifying where consecutive emissions statements are separated by more than two standard-deviations computed from the emissions signal. Having divided the signal into distinct regimes, we produce estimates of a realigned signal by fitting an ensemble of AR(1) random walks, where the relative regime offsets are parameterised. Broadly speaking, this produces estimates close to the long-term trend of the annual emissions signal.

An example of these features is provided by Fig. 11. This example shows how an energy company based in the UK slashed its scope 1 emissions in half by shedding high-intensity electricity generation assets in 2018-2019. This company also illustrates the surprisingly large restatements which can occur and dramatically shift a company’s profile.

### Calculating performance and reduction ambition

Extrapolation and interpolation is our process of estimating emissions of a company which is on track to reach its target at its target date with a uniform reduction factor, and to extrapolate further reductions following the same rate. This is performed with a simple exponential function fit between sequential targets, a common choice of reduction pathway also offered under the SBTi framework^57^. Let $\textrm{target}(t)$ be our modelled target emissions as a fraction of the base year at time *t*, and $r_n$, $d_n$ denote the reduction ambition, and date of the *n*th target respectively. Where $t\le d_1$,

$$\begin{aligned} \log (\textrm{target}(t) + c) = \frac{(t-\text {base~date})\log (1+c-r_1)-(t-d_1)\log (1+c)}{d_1-\text {base~date}} \end{aligned}$$

and for subsequent targets, where $t> d_1$, including beyond the furthest target date,

$$\begin{aligned} \log (\textrm{target}(t) + c) = \frac{(t-d_{n-1})\log (1+c-r_n)-(t-d_n)\log (1+c-r_{n-1})}{d_n-d_{n-1}} \end{aligned}$$

where the constant *c* parameterises the relative bias of reduction rates towards the start of a reduction period. It is not uncommon to see linear interpolation used in corporate models; a fixed year-on-year reduction between targets (corresponding to $c\gg 1$), although anecdotally an exponential-like path appears to be a more common shape both of interim targets, and progress to targets (although here in the early days of target achievement, examples are scarce). Fundamentally, we do not intend to dwell on this precise formulation as the following results are largely insensitive to the approach, fixing $c=0.1$.

## Results

**Fig. 3:**
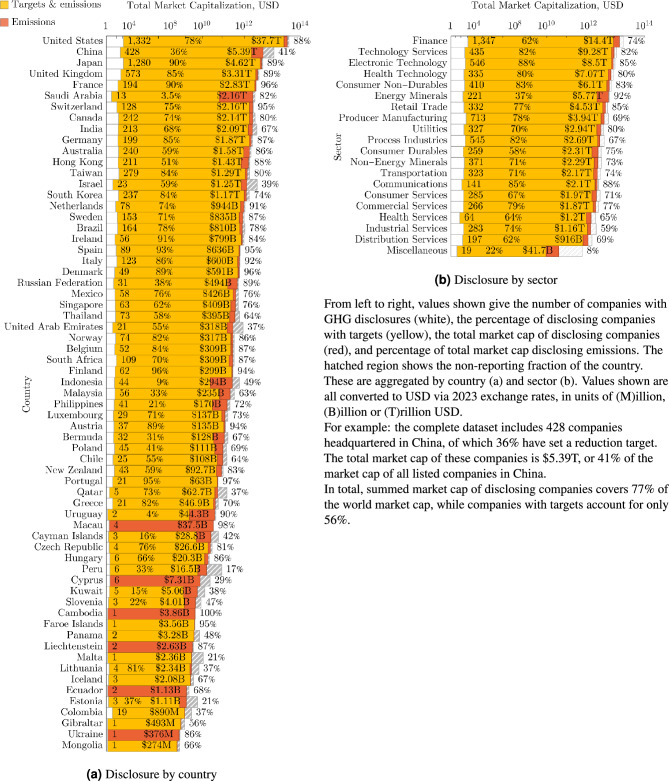
A breakdown of the coverage of our universe in terms of market capitalisation, GHG emissions, and number of disclosing companies.

**Fig. 4:**
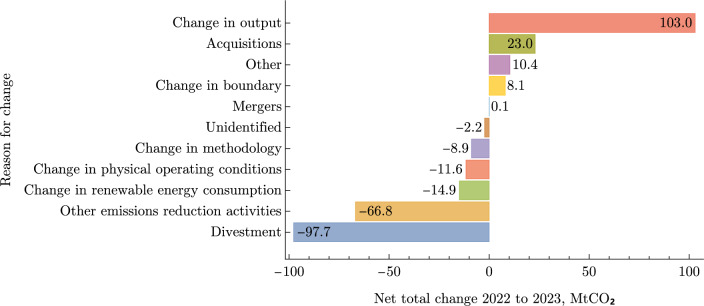
The stated reasons for change in emissions disclosed by companies in the 2022-2023 interval. Emissions are aggregated over companies where the change in emissions accompanying the reasons for change are no more than 25% different from the disclosed emissions over the same period, an error which is introduced by the independence of the two data-entry forms.

The filtered, curated and processed dataset consists of emissions reporting from 7,554 and targets by 3,540 companies globally. The data covers scope 1, 2 and 3 emissions for 7,531, 7,473 and 4,505 companies respectively. Fig. 3a and b show the count and total market cap of disclosing companies, aggregated as a fraction of their registered country and their sector globally. Market cap data is taken for the year 2023 from the FactSet database and is in broad agreement with other sources. Emissions coverage is fairly comprehensive across both individual countries and sectors, in large part due to a concerted effort by the Institutional Shareholder Services (ISS, a supplement to CDP data) to aggregate data which covers most publicly listed companies. Differences between countries and industries are more pronounced in the share of target setting companies. China, along with Middle Eastern and Southeast Asian countries yield a disproportionately low engagement with emissions target setting. Traditionally high GHG intensity industries are also poorly represented in target setting including mineral extraction, process industries, distribution and transportation (Fig. 4).

**Fig. 5:**
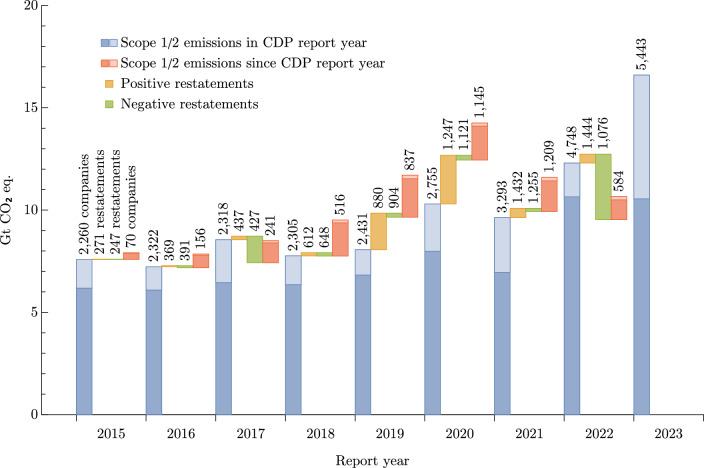
The sum of scope 1 and scope 2 emissions from companies included in CDP annual deliveries of disclosing companies. Each report is supplemented by new companies providing data to CDP which back-fill, and can be modified by restatements in following disclosures. The report year is the timestamp of the disclosed data itself. As companies can backfill their historical emissions, there can be changes in emissions in new disclosures since the report year.

Our company universe consists of 5,998 companies, with a global coverage spanning across 74 countries. We consider two types of breakdowns; in terms of population, and in terms of market capitalisation. To get a better view of each market both need to be considered. For instance, approximately 18% of the number of companies in our dataset is based in the United States of America, however they represent approximately 50% of the market capitalisation covered. Of the 5,998 active companies reporting emissions via CDP, 2,048 make use of restatements with a median of 8 scope 1 and 2 restatements over all disclosures. In aggregate, restatements of past emissions have approximately zero median such that a randomly selected restatement is as likely to be an increase to the previously reported value as it is a decrease. Figure 5 shows the annually aggregated emissions at the reporting year, those back-filled to include this year and the influence of restatements on GHG totals. It includes some instances of large biases in the direction of scope 1 restatements for certain years. The distribution of individual restatements is characteristically fat-tailed, meaning there exist restatements by very large factors.

**Fig. 6:**
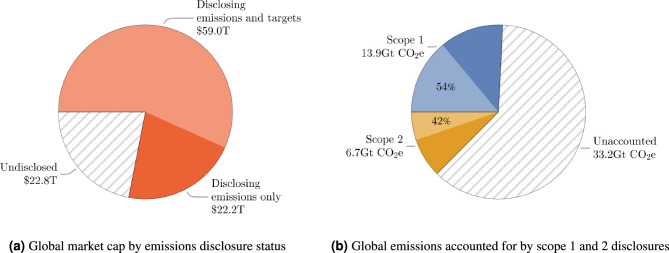
(**a**) Percentage of global market capitalisation by status of emissions disclosure. (**b**) Percentage of global emissions by disclosure category and status. In both cases, the lighter segment indicates the portion of companies with both emissions disclosures and stated targets. While disclosing companies account for a majority of the total market cap globally, they cover substantially less of the global emissions.

Figure 6 shows the aggregated market cap and global GHG emissions covered by disclosing companies, subdivided to show those which disclose targets. Emissions disclosures cover just under 77% of the global public market cap, although the coverage of private markets is likely very small. A broad perspective estimates private capital stock (which excludes capital raised by bonds or debt) to be roughly 250% of public market capitalisation^58^, although this and similar estimates should be understood as a coarse estimate of the relative size of private markets and we do not intend to make inferences about the relative value of public, private and state-owned companies which may be a significant component of the large disparity with the public market coverage and GHG emissions covered. Scope 1 and 2 emissions disclosures account for only 13.9 GtCO${}_2$e (25%) and 6.7 GtCO${}_2$e (13%) of global emissions. Notably the door is left open for double counting; scope 1 emissions of electric utility companies which use carbon-intensive energy sources are counted again in scope 2 emissions of their customers. Rather than attempting to unpick the relationships between reporting companies and their energy providers, we simply aim to estimate the size of this effect. In our dataset, scope 1 emissions of electric utilities come to 3.5 GtCO${}_2$e, a little under 24% of the global total for electricity generation and heating^59^ and therefore a first-order estimate of this effect would discount scope 2 emissions by the same factor, 24%. Were we to expand this study to include scope 3 emissions, this effect would be far more significant and challenging to quantify as both scope 1 and 2 emissions of a company certainly include the use of materials and operation of equipment in its supply chain generated by a company may also be reported by other members of the supply chain in different categories, as for instance in the food sector^60,61^. The need for more sector-specific methodologies for scope 3 accounting methodologies has recognised for some time^62^, and as the coverage of GHG disclosures expands and more emissions are captured under scope 1 for electric utilities rather than scope 2 for consumers, the challenge of double counting will require more thorough treatment.

Another contributor to the discrepancy between market capitalisation and global emissions coverage is scope 3 emissions which are dominated by road transport and residential heating; the International Energy Agency estimates passenger road vehicles to account for 3.5GtCO${}_2$e in 2022^63^, while non-electric residential heating was 2.4Gt^64^, a combined total of approximately 12% of global GHG emissions which would not be covered by scope 1 and 2 corporate emissions.

**Fig. 7:**
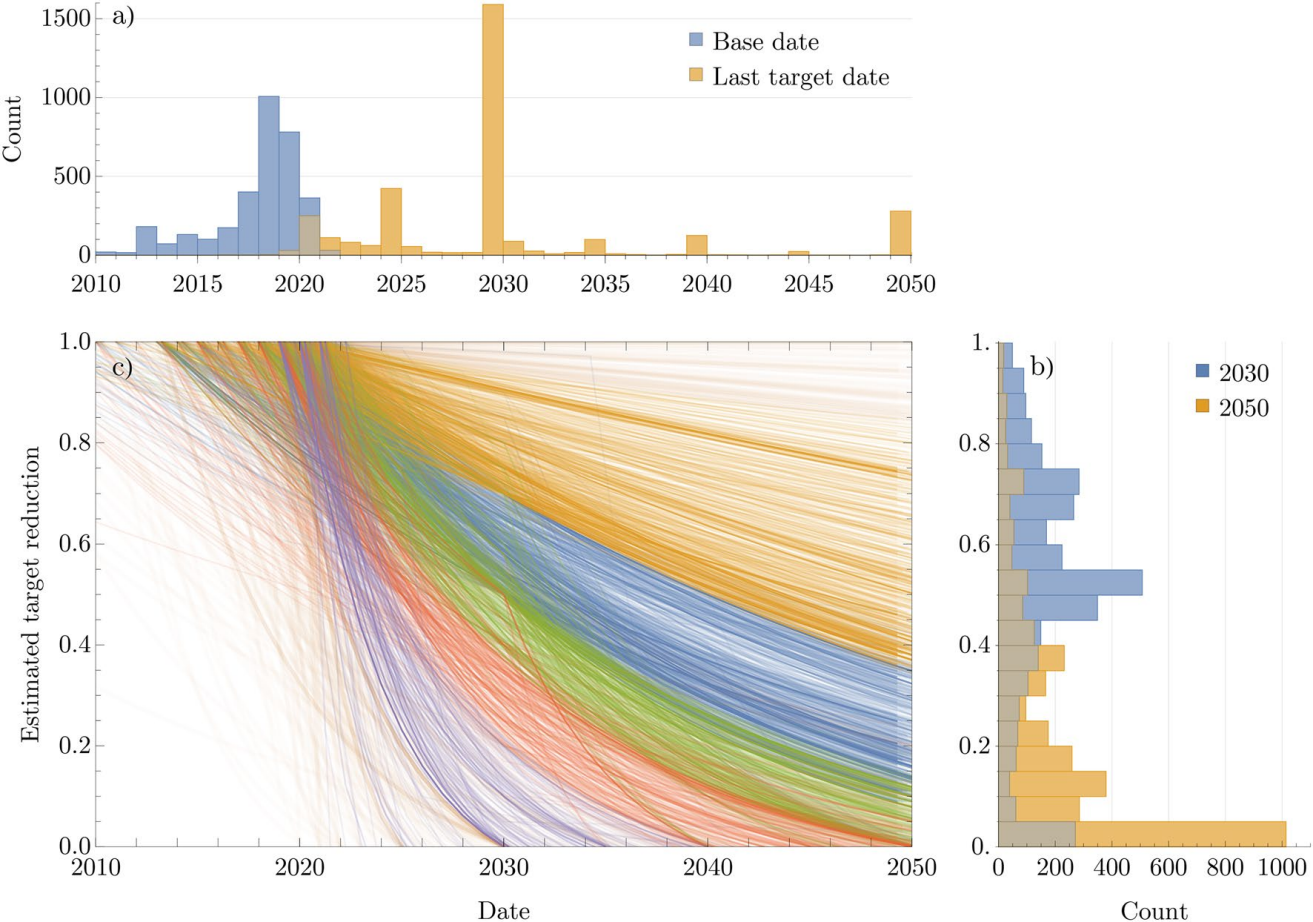
(**a**) The year distribution of base dates and target dates of company emissions reporting and targets. The majority of targets are set no further than 2030, with some very short term 2025 targets and some long-term 2050 targets. (**b**) Reduction ambition interpolated or extrapolated at 2030 and 2050 shows strong modality at 50% reduction in 2030, and 100% reduction in 2050. (**c**) Reduction ambition visualised from each base year, interpolated through stated targets and extrapolated out to 2050 by an exponential function. Targets are taken from both CDP and SBTi. Colours indicate a coarse clustering of the reduction profiles, strongly indicating that companies set targets with significant basis on guidance such as though CDP or SBTi, or their peer groups.

**Fig. 8:**
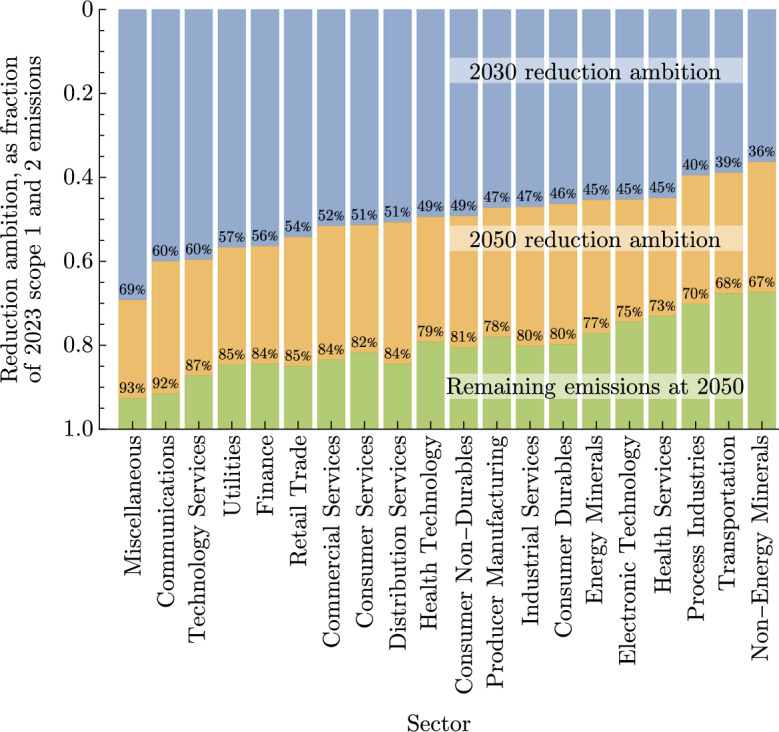
The mean of interpolated and extrapolated reduction ambitions aggregated by sector, ranked by highest to lowest 2030 reduction. For instance, the Commercial Services sector has committed to an 84% reduction of its emissions by 2050, thus leaving 16% unabated. This 84% is achieved first by a 51% reduction by 2030, followed by an additional 33% (84% - 51%) reduction by 2050.

4,505 targets were disclosed by companies to CDP and SBTi which are illustrated by Fig. 7. 2030 emerges as a dominant mid-term milestone, with a corresponding base year typically between 2018-2020. While there is a broad spectrum of reduction ambition, it is not without structure. The colour scheme shown in Fig. 7 results from clustering in the interpolated ambitions, a feature hinted at by the multi-modal shape of the reduction distribution. The least ambitious companies (indicated by purple and yellow) disproportionately fall into the sectors Electronic Technology, Process Industries, Non-Energy Minerals and Transportation. Figure 8 enumerates the mean 2030 and 2050 target values for each sector, showing that the companies estimated to approach net-zero emissions by 2050 (red and blue) generally include service industries such as Commercial and Consumer Services. The most ambitious (green) represent the most easily decarbonised sectors, including Finance, Communications and Technology Services.

**Fig. 9:**
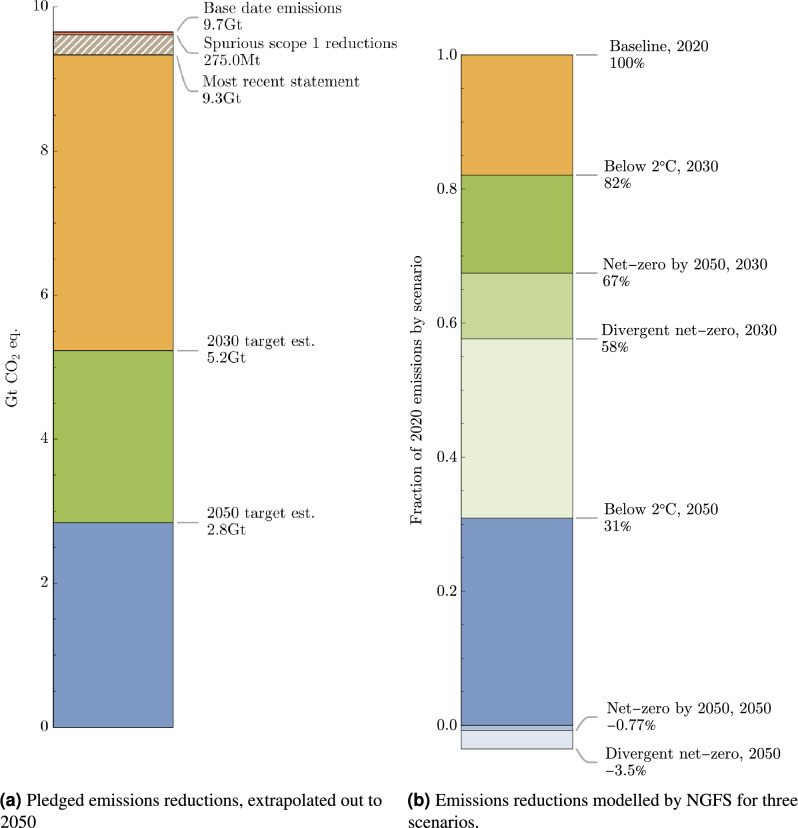
Total emissions disclosures of companies with targets, split into base year emissions, most recent progress, interpolated 2030 reductions and extrapolated 2050 ambitions. Part of the progress since the base year includes spurious reductions to scope 1 emissions (hatched), where two sequential statements are significant outliers bulk of the company emissions signal. These steps account for almost all the reduction since the base years. NGFS scenario decarbonisation is shown for context, showing estimates of global emissions reductions for three scenarios. 2030 company reduction targets roughly align them to the aggressive divergent net-zero scenario at 2030, although a lack of ambitious targets out towards 2050 reduce the 2050 reduction to the less progressive below $2^{\circ }$C scenario.

Figure 9 shows the base year emissions, most recent emissions, interpolated targets at 2030 and extrapolated reductions out to 2050, highlighting the reductions achieved by companies as a result of spurious changes. This is where consecutive emissions disclosures deviate from the company mean by two standard-deviations, and is typically associated with significant changes to methodology such as changing the reporting boundary, or by restructuring such as by divestment. The net effect of these changes to scope 1 emissions are negative and largely responsible for the 3.3% emissions reduction since the base year. Conversely, spurious scope 2 changes are positive, though roughly 25 times smaller in magnitude (10.6 MtCO${}_2$e). Interim targets at 2030 are consistent with the most aggressive decarbonisation scenarios published by NGFS^42^, while towards 2050 extrapolated ambitions are closely aligned to the less optimistic below $2^{\circ }$C scenario.

## Discussion

Our results begin by assessing the development of company emissions reporting as a potential supplement to existing emissions estimation methodologies. We then provide insights into corporate decarbonisation ambition and highlight the importance of identifying and accounting for spurious changes in emissions reporting, such as those due to divestment or methodological shifts, when evaluating progress towards targets. Despite the numerous hurdles discussed, the current level of disclosure, which has grown rapidly since 2020, now provides broad coverage of major companies globally and offers valuable insight into the emissions reduction efforts of the broader economy.

**Fig. 10:**
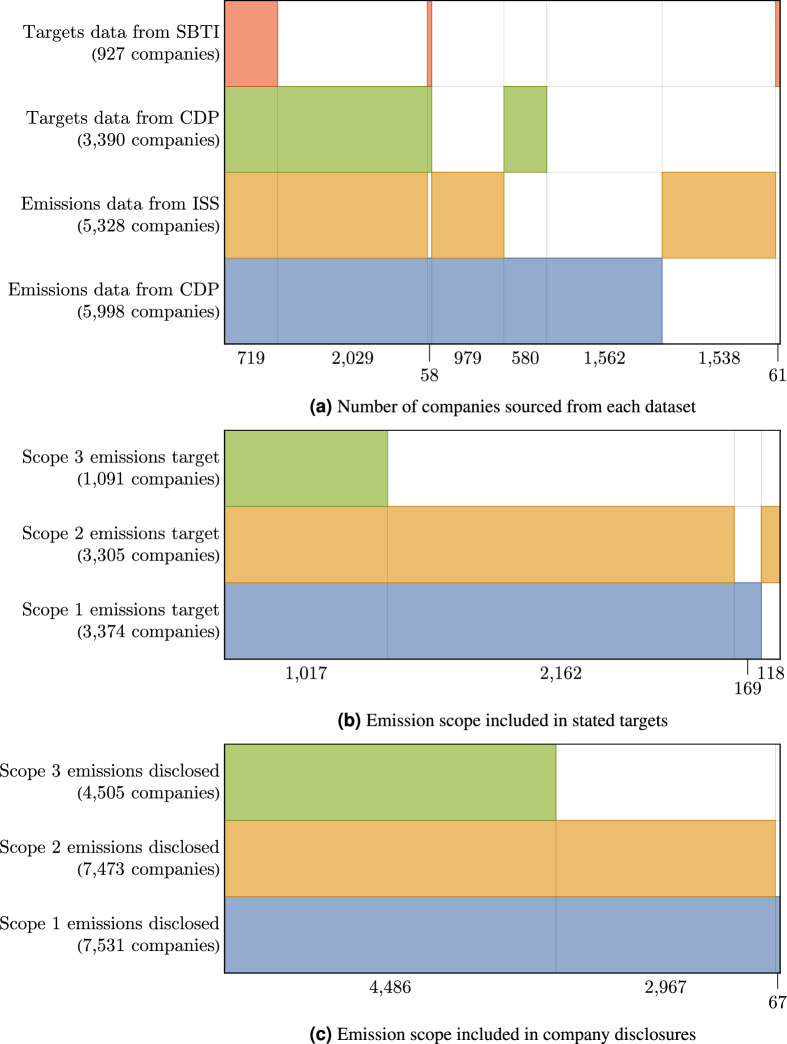
A Venn-diagram, where coloured regions show the intersection of the companies reporting different data through different sources. Where bars overlap vertically, a company reports in multiple datasets or categories. (**a**) companies reporting emissions through the CDP (Carbon Disclosure Project) or ISS (Institutional Shareholder Services), and emissions reduction targets through the CDP or SBTi (Science-Based Targets initiative), (**b**) companies which include scope 1, 2 or 3 emissions in their targets, and (**c**) companies which include scope 1, 2 or 3 emissions in their disclosures. Labels indicate the size of intersection between categories and show a significant amount of overlap between these primary data sources, with CDP covering almost every company with a disclosed target, aside from a small number of additional targets accessible only through SBTi. 60% of companies try to estimate their scope 3 emissions in their disclosures, while 29% set a reduction target based on their scope 3 emissions. 92% of targets cover both scope 1 and 2, with the remainder split roughly evenly between each source.

We show that company reporting has reached a scale and quality sufficient for use, at a global level, as a serious supplement to existing global monitoring schemes. Reported scope 1 emissions now account for approximately 25% of global GHG emissions, and the number of companies disclosing via CDP has nearly doubled since 2020. However, our findings also highlight several important limitations that must be addressed to enhance the utility of company disclosures. A key limitation is the lack of a standardised reporting framework, leading to inconsistencies in the scope and detail of emissions data across companies. This variability makes effective aggregation and comparison difficult (Fig. 10).

A particularly challenging issue is the presence of anomalous step-changes in emissions data, often attributed to divestments. In this study, we have accepted these disclosures without adjustment while tracking their influence on aggregate trends. The result is that nearly all the observed emissions reductions by target-setting companies since their stated base years are explained by such anomalies rather than sustained reductions over time.

Further analysis of reduction ambitions among companies with stated targets revealed clusters of similar ambition profiles, suggesting the emergence of peer-group consensus. This may be driven by the public and often actively advertised nature of emissions targets, encouraging alignment with industry peers. Furthermore, both CDP and SBTi provide frameworks for submitting and evaluating targets. SBTi verifies alignment with the Paris Agreement using decarbonisation models^57^, while CDP typically recommends a constant year-on-year reduction, adjusted for a few GHG-intensive sectors such as cement, aviation, and refinement. These centralised methodologies underscore the role of industry norms and best practices in shaping corporate emissions strategies.

**Fig. 11:**
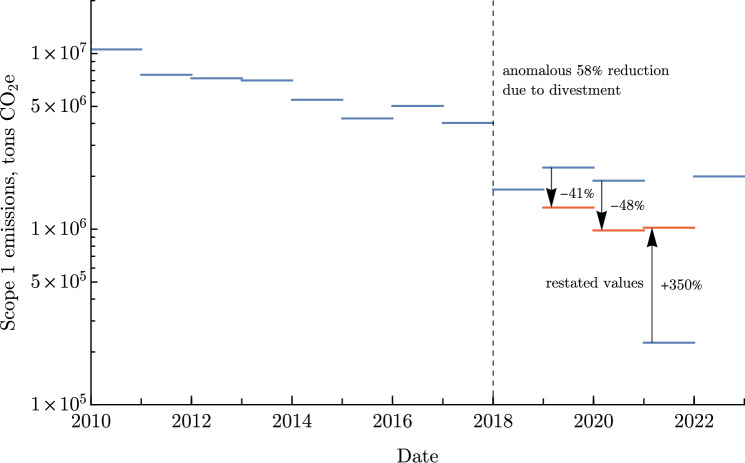
Scope 1 emissions of a large energy company based in the United Kingdom demonstrates two key features of emissions reporting managed in this manuscript. Unprecedented, or anomalous shifts in scope 1 emissions are typically a result of divestment. In this instance, the company sold large fossil fuel assets resulting in a dramatic reduction in scope 1 emissions. Following this, there have been multiple instances of restated emissions, where previous values are replaced in subsequent disclosures.

Encouragingly, some companies, especially in service sectors such as Commercial, Technology, and Consumer Services, have formalised net-zero targets by 2050. However, these industries represent a relatively small share of global emissions. In contrast, high-emitting sectors have yet to commit to similar reductions. As a result, while the short-term targets (to 2030) of disclosing companies align with aggressive decarbonisation scenarios, such as the NGFS divergent net-zero pathway, the long-term outlook remains concerning due to the lack of ambitious 2050 targets among major emitters (Fig. 11).

Although NGFS scenarios include a role for carbon mitigation to achieve net-zero emissions, our analysis has not accounted for corporate offsetting. This is an often controversial practice that involves funding carbon capture, utilisation, and storage (CCUS) and claiming these reductions as part of corporate emissions^65^. Such schemes are not currently part of corporate GHG inventories or the EU Emissions Trading System and are unlikely to scale sufficiently to compensate for the lack of long-term target ambition, with global CCUS capacity for 2030 still under 320 MtCO_2_^66^.

In aggregate, disclosing companies represent nearly 80% of global market capitalisation, yet account for only a small (albeit growing) fraction of total global emissions. This discrepancy raises two key issues. First, it reinforces the urgent need for global reporting standards. Companies failing to disclose emissions or set meaningful targets may be avoiding accountability, an emerging phenomenon termed “green-hushing”^67^. Second, even among disclosing companies, the validity of reported data must be scrutinised to guard against “green-washing”^68,69^. Furthermore, private markets remain largely absent from the CDP dataset, and voluntary expansion seems unlikely. In the absence of comprehensive reporting, it is crucial to assess the expected GHG mitigation burden on private companies, potentially by estimating the industrial composition of the private sector.

## Conclusion

An important takeaway for policymakers is that the proportion of global GHG emissions currently covered by corporate disclosures remains insufficient. We should not assume that current corporate commitments are adequate to deliver the economy-wide reductions required to meet the Paris Agreement goals. Nevertheless, there has been a significant recent acceleration in disclosure coverage, and this momentum, driven by stakeholder pressure^70^, can be amplified by regulatory intervention if necessary.

By engaging proactively with the private sector to improve the coverage, consistency, and accuracy of disclosed emissions, policymakers can gain a valuable bottom-up view of both realised and intended progress on decarbonisation. As demonstrated by our results, such data already provide actionable insights into the sectors most in need of policy support to drive green innovation and curtail emissions-intensive activity.

In expanding GHG reporting, measures to improve data quality, standardisation, and transparency are critical. We observed several notable shifts in reported scope 1 emissions that are not readily explained without considering potential changes in accounting practices or emissions methodologies. These shifts are not necessarily deceptive, but they underscore the need to understand the incentives and disclosure rules that lead to them^69^. Greater regulatory oversight and clearer reporting guidelines can help ensure consistent and comparable disclosures.

This trend coupled with the intensifying emphasis on carbon capture technologies and their strategic implementation in hard-to-abate sectors, may serve as a pivotal driver in expediting the comprehensive decarbonisation of the global economy^71,72^. Concurrently, the growing interest in the use of carbon offsets^73,74^ can further support the transition to a low carbon world, however care should be taken to ensure the quality and resilience of such projects in cases of wildfires, drought, and other exogenous factors^75^.

While this study focused on the stated ambition of emissions reductions, equally important are the emissions produced along the reduction pathway and the feasibility of targets. As climate risk and the transition economy draw increasing attention, there is a growing need for robust data and models to evaluate corporate decarbonisation strategies. Integrating such insights into investment and capital allocation frameworks requires acknowledging the uncertainty and dynamism of real-world companies.

An exponential-like trajectory toward targets reflects a best-case scenario characterised by precisely the kind of progress needed to avoid crossing planetary boundaries^13^. While many companies appear to follow such patterns, others defer reductions to future years despite ambitious targets. Quantifying this behaviour and modelling near-future emissions at the company level will be essential in developing a credible bottom-up approach to inform climate policy and incentives. Such models can serve not only to audit short-term performance but also to assess the realism and sufficiency of long-term targets.

## Data Availability

The aggregated emissions coverage and targets datasets generated and analysed during the study are available from the corresponding author on reasonable request. Disaggregated data is provided by third parties made available under licence that the author does not have permission to share. Emissions data is provided by CDP (www.cdp.net) and ISS (www.issgovernance.com/esg/ratings), emissions targets by CDP and SBTi (www.sciencebasedtargets.org), company financials by FactSet (www.factset.com).

## Glossary

CDP: is a not-for-profit institution that provides a framework for companies to voluntarily disclose information about their GHG emissions. Found at www.cdp.net.
GHG: are greenhouse gases. They trap heat in the atmosphere and drive climate change. They are measured in $CO_{2}$ equivalents, which express each gas’ warming impact relative to carbon dioxide.
ISIN: is the International Securities Identification Number, a 12-digit alphanumeric code that uniquely identifies a specific security, such as a stock or bond. It refers to a financial instrument, not the company itself, though it is commonly used to link back to the issuing company.
ISS: is The Institutional Shareholder Services Inc. is an American advisory firm, allowing clients that own shares of companies to receive advise, and vote on companies’ resolutions.
NGFS: is the Network of Central Banks and Supervisors for Greening the Financial System, launched at the Paris One Planet Summit on 12 December 2017. It is a group of Central Banks and Supervisors willing, on a voluntary basis, to share best practices and contribute to the development of environment and climate risk management in the financial sector and to mobilise mainstream finance to support the transition toward a sustainable economy. Found at ngfs.net.
SBTi: is an initiative that offers companies a clear, actionable path to align emissions reductions with the Paris Agreement goals. Found here https://sciencebasedtargets.org/
